# Supplementary material for: Agricultural products: A study on the impact of social presence on customer engagement in livestream marketing
Source: PLoS One. 2026 Mar 13;21(3):e0342562. doi: 10.1371/journal.pone.0342562 (PMC12987465; doi:10.1371/journal.pone.0342562)
Supplement: S2 File — (DOCX) [file pone.0342562.s002.docx]

Inclusivity in global research

PLOS’ policy on inclusivity in global research aims to improve transparency in the reporting of research performed outside of researchers’ own country or community and ensures that PLOS publications reporting global research adhere to high standards for research ethics and authorship. Authors of relevant research articles may be asked to complete the questionnaire below, which outlines ethical, cultural, and scientific considerations specific to inclusivity in global research. This questionnaire may be requested when researchers have travelled to a different country to conduct research, if research uses samples collected in another country, research with Indigenous populations or their lands, or if research is on cultural artefacts. Researchers travelling to another country solely to use laboratory equipment will not normally be required to complete the questionnaire. However, the questionnaire can be requested at the journal’s discretion for any submission – if you have been requested to complete this questionnaire by the PLOS journal you submitted to, please do so.

Please complete the questionnaire below and include this as a Supporting Information file with your manuscript. Note that if your paper is accepted for publication, this checklist will be published with your article in the supporting information files. Please ensure that you reference the checklist in the main body of your manuscript. We suggest adding a subsection ‘Inclusivity in global research’ to your Methods section and adding the following sentence: “Additional information regarding the ethical, cultural, and scientific considerations specific to inclusivity in global research is included in the Supporting Information (SX Checklist)”

The questions have been designed to be applicable to a wide range of study types, and there are subsections for both human subjects research and non-human subjects research. If any of the questions are not relevant to your research please mark them as “N/A” as appropriate.

**Ethical considerations, permits and authorship**

*This section is applicable to all research types.*

Provide details as to who granted permissions and/or consent for the study to take place in the Methods section of your manuscript. This should include the names of **all** ethics boards, governmental organizations, community leaders or other bodies that provided approval for the study. If individuals provided approval refer to these people by their role or title but do not list their name(s).

Reported on page number: 15

If there were any deviations from the study protocol after approval was obtained please provide details of these changes in the Methods section of your manuscript.
Did this study involve local collaborators that are residents of the country where the research was conducted or members of the community studied? If you do not have any authors from said communities, please provide an explanation for this below.

Reported on page number: N/A

Yes, all authors of this study are residents and academic researchers based in China, the country where the research was conducted and from which the study sample was drawn. This was a domestic study conducted within our own national context, and all participants were part of the broader national consumer community under investigation.

Everyone listed as an author should meet PLOS’ criteria for authorship and all individuals who meet these criteria should be included in the author byline, rather than the acknowledgements. For further information please see the journal’s Authorship Policy.

**Human subjects research (e.g. health research, medical research, cross-cultural psychology)**

Did you obtain written informed consent from a representative of the local community or region before the research took place? How did you establish who speaks for the community? Details of written informed consent obtained from study participants should be reported separately in the Methods section of your manuscript.

No. Written informed consent from a formal representative of a local community or region was neither sought nor required for this study.

The “community” under study is the national population of consumers in China who engage with agricultural product livestream marketing. This is a large, dispersed demographic group without a defined geographical boundary, traditional governance structure, or single appointed spokesperson. Therefore, the concept of identifying a sole community representative for granting prior research permission is not applicable to this research context.

As reported in the Methods section (Manuscript, page 15), the process for obtaining individual consent was as follows:

“This questionnaire-based study was conducted in accordance with ethical principles for research involving human participants. Participation was voluntary, and written informed consent was obtained from all participants prior to participation.”

How did members of the local community provide input on the aims of the research investigation, its methodology, and its anticipated outcome(s)?

When engaging with the local community, how did you ensure that the informed consent documents and other materials could be understood by local stakeholders?

Given that the “community” under study is a national population of consumers, formal collaborative design with community representatives was not applicable. Community input was integrated indirectly through:

Basing the research questions on prior literature about consumer behavior.

Collecting direct data from survey, which forms the core evidence for the study.

Aligning the research with practical issues in the livestream marketing ecosystem that affect consumers.

Thus, the community’s perspective is central to the study’s empirical basis and relevance.

The primary local stakeholders in this study were the individual consumer participants. To ensure all research materials were fully understandable to them, the following steps were taken:

1. Language Accessibility: All study materials, including the digital informed consent statement and the questionnaire, were designed and presented in Standard Mandarin Chinese, the primary language of the target participant population.
2. Clarity and Simplicity: The consent statement and survey items were drafted using clear, non-technical language appropriate for a general adult audience.
3. Contextual Delivery: The consent information was presented at the very beginning of the online survey on the Wenjuanxing platform. Participants had to read and acknowledge this information before they could proceed to the questionnaire, ensuring exposure to the consent terms.

Since the research did not involve engagement with a specific, bounded community requiring cultural translation or liaison with community leaders, no further adaptation of materials was necessary. Comprehension was ensured by aligning the materials directly with the participants' linguistic and cultural context.

Will the findings of the research be made available in an understandable format to stakeholders in the community where the study was conducted (e.g. via a presentation, summary report, copies of publications, etc.)? Please provide details of how this will be achieved.

Yes. The findings of this study will be made publicly available through publication in an open-access academic journal. This ensures that relevant stakeholders, including consumers, practitioners, and researchers interested in agricultural livestream marketing, can access the results in an understandable format. The published article will present the findings using clear language, tables, and figures to facilitate comprehension.

**Non-human subjects research using specimens/ animals collected as part of the study, or those housed in archival collections. Examples include archaeology, paleontology, botany and zoology.**

Did the permission you obtained from a local authority to perform the study include an agreement on access to outputs and benefit sharing? This may include procedures to enable fair distribution of the benefits and resources arising from the research performed. Please include any details of Prior Informed Consent and Benefit Sharing Agreements obtained. These may be required by field-specific regulations, for example the Convention on Biological Diversity (CBD) and the associated Nagoya Protocol.

N/A

If the material used in your study was imported, please A) provide the year it was imported and B) indicate whether permits were obtained to import/export the materials used, C) provide details of any permits obtained. If this information is not available, please indicate this.

N/A

If you used archival specimens, please state how the material used in your study was acquired by the institute it is held in and provide details of any permits obtained for the original excavations/ sample collection. If this information is not available, please indicate this.

N/A

How was the potential cultural significance of the materials collected in your study to local communities considered in your research design? Were Indigenous peoples and/or local researchers and institutions involved with archaeological excavations / collection of specimens? If so, please provide a description of their involvement.

N/A

If your manuscript includes photographs of human remains please indicate whether authors obtained permission from descendants or affiliated cultural communities to do so.

N/A
