# Supplementary material for: Agricultural products: A study on the impact of social presence on customer engagement in livestream marketing
Source: PLoS One. 2026 Mar 13;21(3):e0342562. doi: 10.1371/journal.pone.0342562 (PMC12987465; doi:10.1371/journal.pone.0342562)
Supplement: Attachment — Submitted filename: PONE-D-25-56485_reviewer report.pdf [file pone.0342562.s003.pdf]

Reviewer Report

Paper Title: Agricultural Products Study on the Impact of Social Presence on Customer Engagement in Livestream Marketing

Manuscript Number: PONE-D-25-56485

Reviewer Name: Dr Mouza Al Hadhrami
OrcID: 0000-0003-1288-9591

Thank you for inviting me to review this paper; I found it interesting and academically sound. Please see my comments below for improvement.

1. **The Abstract**

Well-structured and decent. My only concern is that it should avoid repetition. The abstract repeats the idea of social presence promoting customer engagement multiple times. Consider streamlining this to avoid redundancy. Also, the practical recommendations are valuable but could be summarized more succinctly. For example, "**The study provides actionable insights for optimizing agricultural livestream marketing, including fostering trust, enhancing interactivity, and supporting farmers and e-commerce platforms**."

2. **Introduction:**

The introduction section follows a standard academic structure, providing a clear context, identifying gaps in existing research, and outlining the study's objectives and contributions. Main enhancement areas might be:

- Lack of a clear research problem statement. Just be more explicit and straightforward, emphasizing that the specific mechanisms by which social presence, customer satisfaction, and trust climate influence customer engagement **remain underexplored.**
- Need to highlight the novelty by emphasizing that the **study is among the first to integrate social presence and trust climate into the consumer behaviour framework, providing a fresh perspective on livestream marketing for agricultural products** (if I am not mistaken).
- The section mentions the limitations of traditional consumer behavior theories, but could explicitly highlight how the study addresses these gaps (paragraph 8)

3. **Theory and Research Hypotheses**

I prefer to add “literature review” as a main headline with a brief introduction on how you are about to discuss the Theory and Research Hypotheses. Never mind, if this is done according to the journal template.

This part has a clear structure, effectively incorporates prior research to support the hypotheses, which are found clearly and logically derived from the discussion. Here are my points of concern:

- The hypotheses are well-stated, but the rationale for each could be expanded slightly in one paragraph under each one. For example:
  - For H1, explain why social presence is particularly impactful in the agricultural product sector compared to other industries.
  - For H2, elaborate on how customer satisfaction mediates the relationship between social presence and engagement, perhaps with a brief example or scenario.
  - For H3 and H4, provide more detail on why trust climate is especially critical in agricultural product marketing (e.g., the nature of agricultural products requiring trust due to their perishability and quality variability).
  - One more point is to conclude this subsection by briefly mentioning how the hypotheses contribute to advancing consumer behavior theory in the context of digital marketing

- In Figure 3, Research Model: I would recommend including arrows with labels to indicate the type of relationship (e.g., "positive impact," "moderating effect")

4. **Research Design:**

   Areas for improvement:

- **Lack of Sampling Method**: The section mentions the use of the Wenjuanxing survey platform and the distribution of 350 electronic questionnaires, but it does not specify the sampling method (e.g., random sampling, convenience sampling, stratified sampling). This is crucial for understanding the representativeness of the sample.
- **Data Collection Process**: The section does not provide details about how the data was collected (e.g., duration of the survey, methods to ensure data quality, or any incentives provided to respondents).
- The section does not mention any ethical considerations, such as informed consent or data privacy measures, which are critical for research involving human participants.

5. Empirical Analysis

An explicit link between the finding and the theoretical concepts discussed earlier (e.g., user value theory, value-satisfaction causal theory, trust theory) is a must.

- Under 4.3: hypothesis testing: Explicitly connect the empirical findings to the theoretical framework. Here are two examples:
    - "The significant impact of social presence on customer engagement behavior (H1) aligns with the findings of Huang et al. (2023) (**paragraph 1 in 4.3 section**), who emphasized the role of social presence in influencing consumer behavior during livestreaming." **(Paragraph 1 in 2.1 Social Presence and Customer Engagement Behavior)**
    - "The mediating role of customer satisfaction (H2) supports the value-satisfaction causal theory proposed by Woodruff (1997), which suggests that satisfaction is a key driver of customer behavior." (**paragraph 1 in 2.2 The Mediating Role of Customer Satisfaction)**

- You can also link the analysis to some references you mentioned above.
- Add a summary of the key findings for each table.

6. Main Conclusions and Policy Implications

- Well-written and academically sounding discussion and recommendations. However, I would consider simplifying the titles to make them more direct and appealing. For example:
  5.1 Key Findings
  5.2 Theoretical Contributions
  5.3 Policy Recommendations
  5.4 Limitations and Future Research
- In the Contribution to the Literature, you need to link it to the theories to explain how this contribution advances the field. For example, you argued in the literature review that real-time interaction and emotional resonance play a critical role in customer decision-making during livestreaming marketing (paragraph 6 in the Introduction). Your contribution underscores the need for a more dynamic, interactive framework to understand consumer behavior in digital marketing contexts (paragraph 1 in the contribution to the literature).

- For the **Policy Implications**, try to rank the recommendations based on their importance or feasibility, and explain why specific recommendations should be prioritized. Moreover, the section does not address potential implementation challenges or barriers (e.g., a lack of digital literacy among farmers and infrastructure limitations in rural areas).

I hope my comments are going to receive careful attention and contribute to the improvement of the paper.
