# Supplementary material for: Agricultural products: A study on the impact of social presence on customer engagement in livestream marketing
Source: PLoS One. 2026 Mar 13;21(3):e0342562. doi: 10.1371/journal.pone.0342562 (PMC12987465; doi:10.1371/journal.pone.0342562)
Supplement: Attachment — Submitted filename: Response to Reviewers.docx [file pone.0342562.s005.docx]

**Response to Reviewers**

**Manuscript Title:**

*Agricultural products: A study on the impact of social presence on customer engagement in livestream marketing*

**Manuscript ID:** PONE-D-25-56485

**Journal:** *PLOS ONE*

**Dear Editor and Reviewers,**

We sincerely thank you and the reviewers for the time and effort devoted to reviewing our manuscript. We are grateful for the insightful and constructive comments, which have greatly helped us to improve the quality, clarity, and rigor of our study.

We have carefully revised the manuscript in response to all comments and suggestions. All changes have been highlighted in the revised manuscript using track changes. Below, we provide a detailed, point-by-point response to each comment. For clarity, reviewers’ comments are reproduced in bold, followed by our responses.

We hope that the revisions adequately address the concerns raised and that the revised manuscript will be suitable for publication in *PLOS ONE*.

Once again, we sincerely appreciate the editor’s and reviewers’ valuable feedback.

Kind regards,

Qiheng Sun, Jinying Cao, Chen Guo, Yuanhao Shi

Corresponding author: Jinying Cao

E-mail: caojy@qhit.edu.cn

**Response to Editor’s Comments**

**Editor’s Comment 1:**

Please ensure that your manuscript meets PLOS ONE's style requirements, including those for file naming.

**Response:**

Thank you very much for this reminder. We have carefully revised the manuscript to ensure full compliance with PLOS ONE’s style and formatting requirements. Specifically, we have reformatted the main text, title page, author affiliations, and file naming according to the official PLOS ONE templates provided by the journal. We confirm that the revised submission now adheres to all relevant PLOS ONE formatting guidelines.

**Editor’s Comment 2:**

Please include a complete copy of PLOS’ questionnaire on inclusivity in global research in your revised manuscript. Our policy for research in this area aims to improve transparency in the reporting of research performed outside of researchers’ own country or community. The policy applies to researchers who have travelled to a different country to conduct research, research with Indigenous populations or their lands, and research on cultural artefacts. The questionnaire can also be requested at the journal’s discretion for any other submissions, even if these conditions are not met. Please upload a completed version of your questionnaire as Supporting Information when you resubmit your manuscript.

**Response:**

Thank you very much for this important comment and for drawing our attention to PLOS ONE’s policy on inclusivity in global research. We have carefully completed the PLOS questionnaire on inclusivity in global research and included a full copy in the revised submission. The completed questionnaire has been uploaded as Supporting Information and is also referenced in the manuscript on pages 34–38. We confirm that this revision complies with the journal’s transparency and reporting requirements.

**Editor’s Comment 3:**

Please provide additional details regarding participant consent. In the ethics statement in the Methods and online submission information, please ensure that you have specified (1) whether consent was informed and (2) what type you obtained (for instance, written or verbal, and if verbal, how it was documented and witnessed). If your study included minors, state whether you obtained consent from parents or guardians. If the need for consent was waived by the ethics committee, please include this information.

**Response:**

Thank you very much for this important comment regarding ethical considerations and participant consent. In response, we have revised the manuscript to explicitly clarify the nature and type of consent obtained from participants. Specifically, in Section 3.1 Sample and Data (paragraph 2-3), we have added a statement indicating that participation was voluntary and that written informed consent was obtained from all participants prior to participation. This revision can be found in the revised manuscript in Section 3.1.

**Editor’s Comment 4:**

Thank you for stating the following financial disclosure:

This research was funded by “Kunlun Talent” Introduction Research Project of Qinghai Institute of Technology, grant number 2023-QLGKLYCZX-030.

Please state what role the funders took in the study. If the funders had no role, please state: "The funders had no role in study design, data collection and analysis, decision to publish, or preparation of the manuscript."

If this statement is not correct you must amend it as needed.

**Response:**

Thank you very much for this comment. In accordance with the editor’s request, we have clarified the Role of the Funder statement. Specifically, we have added the amended statement in the Cover Letter (paragraph 5), indicating that the funder provided access to databases and electronic library resources, as well as financial support for the conduct of the study, but had no role in the study design, data collection and analysis, decision to publish, or preparation of the manuscript. We appreciate the editor’s assistance in updating the online submission form accordingly.

**Editor’s Comment 5:**

We note that you have indicated that there are restrictions to data sharing for this study. PLOS only allows data to be available upon request if there are legal or ethical restrictions on sharing data publicly. Before we proceed with your manuscript, please address the following prompts: a) If there are ethical or legal restrictions on sharing a de-identified data set, please explain them in detail (e.g., data contain potentially identifying or sensitive patient information, data are owned by a third-party organization, etc.) and who has imposed them (e.g., a Research Ethics Committee or Institutional Review Board, etc.). Please also provide contact information for a data access committee, ethics committee, or other institutional body to which data requests may be sent. b) If there are no restrictions, please upload the minimal anonymized data set necessary to replicate your study findings to a stable, public repository and provide us with the relevant URLs, DOIs, or accession numbers.You also have the option of uploading the data as Supporting Information files, but we would recommend depositing data directly to a data repository if possible.

We will update your Data Availability statement on your behalf to reflect the information you provide.

**Response:**

Thank you very much for this important comment regarding data availability. In response, we have revised our data sharing approach to ensure full compliance with PLOS ONE’ s data availability policy. There are no legal or ethical restrictions on sharing the data underlying this study. Accordingly, we have uploaded the minimal anonymized dataset necessary to replicate the study findings as Supporting Information files in the revised submission. All data have been fully de-identified prior to sharing. We believe this revision adequately addresses the journal’ s data availability requirements.

**Editor’s Comment 6:**

We note that your Data Availability Statement is currently as follows: All relevant data are within the manuscript and its Supporting Information files.

Please confirm at this time whether or not your submission contains all raw data required to replicate the results of your study. Authors must share the “minimal data set” for their submission. PLOS defines the minimal data set to consist of the data required to replicate all study findings reported in the article, as well as related metadata and methods.

For example, authors should submit the following data:

-The values behind the means, standard deviations and other measures reported;

-The values used to build graphs;

-The points extracted from images for analysis.

**Response:**

Thank you very much for this clarification request. We confirm that the revised submission includes the complete minimal dataset required to replicate all findings reported in the study. Specifically, the Supporting Information files contain the raw questionnaire response data underlying all descriptive statistics presented in the manuscript.

**Editor’s Comment 7:**

If the reviewer comments include a recommendation to cite specific previously published works, please review and evaluate these publications to determine whether they are relevant and should be cited. There is no requirement to cite these works unless the editor has indicated otherwise.

**Response:**

Thank you for this clarification. The reviewer comments did not include recommendations to cite specific previously published works. Therefore, no additional citations were added in response to this comment.

**Response to Reviewer #1**

**Comment 1:**

The paper claims to extend consumer behavior theory, but existing work on livestream commerce, social presence, and trust is already substantial. The authors should clarify what is novel relative to recent livestream commerce studies (e.g., SOR frameworks, presence-trust-engagement models).

**Response:**

We sincerely thank the reviewer for this important and constructive comment. We fully agree that prior research on livestream commerce, social presence, and trust—particularly studies based on SOR frameworks and presence–trust–engagement models—has established a solid theoretical foundation. To more clearly articulate the novelty of our study relative to this existing literature, we have revised the Introduction (paragraph 9) to explicitly highlight our Novelty.

**Comment 2:**

The sample (312 respondents) is reasonable, but the sampling method (Wenjuanxing) suggests a convenience sample. Potential sampling bias should be acknowledged. It will be helpful if the authors could provide additional demographic characteristics (e.g., platform usage frequency, livestream categories used).

**Response:**

We sincerely thank the reviewer for this insightful comment. We would like to clarify that the study used a snowball sampling method rather than a simple convenience sample. Specifically, data were collected via the Wenjuanxing survey platform using snowball sampling, which allowed us to reach a sufficiently large and relevant participant pool engaged in agricultural livestreaming.

We have clarified the sampling method in the revised manuscript (Section 3.1 Sample and Data, first paragraph, lines 295–296).

**Comment 3:**

Measurement items are described but not presented in a full instrument table. Many journals require a complete item list in Appendix.

**Response:**

Thank you very much for this helpful comment. We have uploaded the full questionnaire and minimal anonymized dataset, as Supporting Information files in the revised submission.

**Comment4:**

While results support hypotheses, theoretical explanations could be expanded.

**Response:**

We sincerely thank the reviewer for this valuable suggestion. In response, we have expanded the theoretical explanations for each hypothesis in Section 2 Literature Review and Research Hypotheses. Specifically, we have provided more detailed reasoning and literature support for the relationships between social presence, customer satisfaction, trust climate, and customer engagement in agricultural livestreaming contexts. We believe these revisions strengthen the theoretical grounding of the study and provide a clearer rationale for the proposed hypotheses.

**Response to Reviewer #2**

**Comment 1: The Abstract**

Well-structured and decent. My only concern is that it should avoid repetition. The abstract repeats the idea of social presence promoting customer engagement multiple times. Consider streamlining this to avoid redundancy. Also, the practical recommendations are valuable but could be summarized more succinctly. For example, "The study provides actionable insights for optimizing agricultural livestream marketing, including fostering trust, enhancing interactivity, and supporting farmers and e-commerce platforms."

**Response:**

We sincerely thank the reviewer for this helpful and constructive suggestion. In response, we have streamlined the abstract to reduce redundancy regarding social presence and customer engagement. Additionally, we have incorporated the reviewer’s suggested sentence to succinctly summarize the practical recommendations. The entire abstract has been language-polished and revised for clarity and conciseness. These changes can be found in the revised manuscript on page 2.

**Comment 2: Introduction**

The introduction section follows a standard academic structure, providing a clear context, identifying gaps in existing research, and outlining the study's objectives and contributions. Main enhancement areas might be:

(1) Lack of a clear research problem statement. Just be more explicit and straightforward, emphasizing that the specific mechanisms by which social presence, customer satisfaction, and trust climate influence customer engagement remain underexplored.
(2) Need to highlight the novelty by emphasizing that the study is among the first to integrate social presence and trust climate into the consumer behaviour framework, providing a fresh perspective on livestream marketing for agricultural products (if I am not mistaken).
(3) The section mentions the limitations of traditional consumer behavior theories, but could explicitly highlight how the study addresses these gaps (paragraph 8)

**Response:**

(1) Thank you for this valuable suggestion. We agree that the research problem statement needed to be stated more explicitly and directly. In response, we have revised the Introduction to clearly highlight the existing research gap. Specifically, we now emphasize that while social presence has been widely examined in livestream marketing, the specific mechanisms through which it influences customer engagement remain insufficiently understood. Moreover, we explicitly point out the underexplored mediating role of customer satisfaction and the moderating role of trust climate in highly interactive livestream environments.

These revisions have been added in the Introduction (Page 6-7, Paragraph 8, Lines 119–122).

(2) Thank you for this positive and encouraging comment. In response, we have explicitly articulated the theoretical contribution and innovation of this study in the manuscript. Specifically, we have added a clear statement highlighting the development of an integrative theoretical framework that incorporates social presence, customer satisfaction, and trust climate, and emphasized its theoretical and practical significance in the context of agricultural livestream marketing.

These revisions have been added in the Introduction (Page 7, Paragraph 9).

(3) Thank you for this helpful suggestion. We agree that the original text could more clearly explain how the proposed study addresses the limitations of traditional consumer behavior theories. In response, we have revised Paragraph 8 of the Introduction to explicitly describe how the integration of social presence and trust climate within a unified framework addresses these gaps. In particular, we clarify the use of a serial mediation model to explain how livestream marketing transforms product information into a credible and empathetic experience through enhanced interaction and trust, thereby extending the theoretical boundaries of traditional consumer behavior theories in livestream contexts.

These revisions have been added in the Introduction (Page 7, Paragraph 8, Lines 124–128).

**Comment 3: Theory and Research Hypotheses**

1.I prefer to add “literature review” as a main headline with a brief introduction on how you are about to discuss the Theory and Research Hypotheses. Never mind, if this is done according to the journal template.

2.This part has a clear structure, effectively incorporates prior research to support the hypotheses, which are found clearly and logically derived from the discussion. Here are my points of concern:

(1)The hypotheses are well-stated, but the rationale for each could be expanded slightly in one paragraph under each one. For example:

For H1, explain why social presence is particularly impactful in the agricultural product sector compared to other industries.

For H2, elaborate on how customer satisfaction mediates the relationship between social presence and engagement, perhaps with a brief example or scenario.

For H3 and H4, provide more detail on why trust climate is especially critical in agricultural product marketing (e.g., the nature of agricultural products requiring trust due to their perishability and quality variability).

One more point is to conclude this subsection by briefly mentioning how the hypotheses contribute to advancing consumer behavior theory in the context of digital marketing

(2)In Figure 3, Research Model: I would recommend including arrows with labels to

indicate the type of relationship (e.g., "positive impact," "moderating effect")

**Response:**

1.Thank you for this suggestion. In response, we have revised the section heading to “2. Literature Review and Research Hypotheses” to more clearly reflect the structure and content of this section and with a brief introduction on how we are about to discuss the Theory and Research Hypotheses.

2(1).Thank you for this insightful and constructive suggestion. We agree that providing a more detailed theoretical rationale for each hypothesis would strengthen the clarity and theoretical grounding of the study. In response, we have substantially expanded the theoretical explanations underlying each hypothesis.

Specifically, in **Section 2.1**, we added a dedicated paragraph explaining why social presence is particularly influential in agricultural product livestream marketing, emphasizing product quality uncertainty, information asymmetry, and the role of immersive, real-time interaction in reducing these challenges.

In **Section 2.2**, we expanded the second paragraph to elaborate on the mediating role of customer satisfaction, illustrating how social presence enhances satisfaction through improved information transparency, emotional interaction, and service responsiveness, which in turn motivates deeper customer engagement.

In **Section 2.3**, we enriched the theoretical discussion in the second and sixth paragraphs to clarify why trust climate is especially critical in agricultural product marketing. The revisions highlight the perishability, quality variability, and experience-based nature of agricultural products, explaining how a high-trust climate strengthens both the direct effect of social presence on engagement (H3) and the mediating role of customer satisfaction (H4).

Finally, we added a concluding paragraph to this subsection summarizing how the proposed hypotheses collectively advance consumer behavior theory by extending traditional engagement models into interactive, trust-dependent digital marketing contexts, with a specific focus on livestream commerce.

These revisions can be found in the Literature Review and Research Hypotheses section (Sections 2.1–2.3).

2(2).Thank you for this helpful suggestion. In response, we have revised Figure 3 to include directional arrows with explicit labels indicating the nature of each relationship, such as positive effects, mediating effects, and moderating effects (H3 and H4). These revisions improve the clarity and interpretability of the research model.

**Comment 4: Research Design**

Areas for improvement:

(1)Lack of Sampling Method: The section mentions the use of the Wenjuanxing survey platform and the distribution of 350 electronic questionnaires, but it does not specify the sampling method (e.g., random sampling, convenience sampling, stratified sampling). This is crucial for understanding the representativeness of the sample.

(2)Data Collection Process: The section does not provide details about how the data was collected (e.g., duration of the survey, methods to ensure data quality, or any incentives provided to respondents).

(3)The section does not mention any ethical considerations, such as informed consent or data privacy measures, which are critical for research involving human participants.

**Response:**

(1) Thank you for these important methodological suggestions. In response, we have revised the **Research Design** section to provide clearer details regarding the sampling method and data collection process. Specifically, we clarify that this study employed a **snowball sampling method** to recruit respondents with experience in agricultural product livestream marketing. In addition, we specify that the data collection was conducted via the Wenjuanxing survey platform over a period of approximately **two months, from April to June 2025**.
(2) Thank you for raising this important point. In response, we have revised Section 3.1 (Sample and Data) to explicitly address ethical considerations. We now state that the study was conducted in accordance with ethical principles for research involving human participants, that participation was voluntary, and that written informed consent was obtained from all participants prior to participation.

This information has been added on Page 15, Section 3.1.

**Comment 5: Empirical Analysis**

An explicit link between the finding and the theoretical concepts discussed earlier (e.g., user value theory, value-satisfaction causal theory, trust theory) is a must.

(1)Under 4.3: hypothesis testing: Explicitly connect the empirical findings to the theoretical framework. Here are two examples:

"The significant impact of social presence on customer engagement behavior (H1) aligns with the findings of Huang et al. (2023) (paragraph 1 in 4.3 section), who emphasized the role of social presence in influencing consumer behavior during livestreaming." (Paragraph 1 in 2.1 Social Presence and Customer Engagement Behavior)

"The mediating role of customer satisfaction (H2) supports the value-satisfaction causal theory proposed by Woodruff (1997), which suggests that satisfaction is a key driver of customer behavior." (paragraph 1 in 2.2 The Mediating Role of Customer Satisfaction)

(2)You can also link the analysis to some references you mentioned above.

(3)Add a summary of the key findings for each table.

**Response:**

(1) Thank you for this important and constructive suggestion. We agree that a clearer linkage between the empirical findings and the theoretical framework strengthens the interpretability and theoretical contribution of the study. In response, we have revised **Section 4.3 (Hypothesis Testing)** to explicitly connect each key empirical result to the corresponding theoretical perspectives discussed earlier in the manuscript.

Specifically, we now relate the significant effect of social presence on customer engagement (H1) to prior research on social presence in livestream commerce (Huang et al., 2023), reinforcing its theoretical foundation. We further link the mediating role of customer satisfaction (H2) to the **value-satisfaction causal theory** (Woodruff, 1997), highlighting satisfaction as a key behavioral driver. In addition, the moderating effects of trust climate (H3 and H4) are explicitly discussed in relation to **trust theory** (Morgan & Hunt, 1994), with particular emphasis on the high uncertainty and experience-based nature of agricultural products.

These theory-driven interpretations have been added throughout the four paragraphs of **Section 4.3**, ensuring a clearer and more coherent connection between the empirical results and the theoretical framework.

(2) We thank the reviewer for the valuable suggestion. In response, we have revised **Section 4 (Empirical Analysis)** by incorporating relevant references mentioned earlier in the manuscript, in order to better link our analysis with the existing literature.
(3) We thank the reviewer for the helpful suggestion regarding the inclusion of key findings for each table. In response, we have added concise summaries of the main results for all tables as follows:

- **Table 1 (Combined Reliability and Convergent Validity Tests)** – The measurement scales for all variables demonstrated good internal consistency, convergent validity, and discriminant validity, with the overall model fit reaching acceptable standards (Section 4.1, first paragraph, lines 348-351).
- **Table 2 (Hierarchical Regression Test Results)** – The results indicate that social presence significantly promotes both customer engagement and customer satisfaction, while trust climate plays a significant moderating role in this mechanism (Section 4.3, first paragraph, lines 388–391).
- **Table 3 (Bootstrap Test Results for Mediation)** – The results show that social presence not only directly affects customer engagement but also exerts an indirect influence through customer satisfaction, supporting the presence of partial mediation (Section 4.3, second paragraph, lines 414–417).
- **Table 4 (Bootstrap Test Results for Moderated Mediation)** – The moderated mediation results indicate that the indirect effect of social presence on customer engagement via customer satisfaction is stronger under a high trust climate, highlighting the contextual importance of trust in agricultural livestream marketing (Section 4.3, fourth paragraph, lines 435–438).

**Comment 6: Main Conclusions and Policy Implications**

1.Well-written and academically sounding discussion and recommendations. However, I would consider simplifying the titles to make them more direct and appealing. For example:

5.1 Key Findings

5.2 Theoretical Contributions

5.3 Policy Recommendations

5.4 Limitations and Future Research

2.In the Contribution to the Literature, you need to link it to the theories to explain how this contribution advances the field. For example, you argued in the literature review that real-time interaction and emotional resonance play a critical role in customer decision-making during livestreaming marketing (paragraph 6 in the Introduction). Your contribution underscores the need for a more dynamic, interactive framework to understand consumer behavior in digital marketing contexts (paragraph 1 in the contribution to the literature).

3.For the Policy Implications, try to rank the recommendations based on their importance or feasibility, and explain why specific recommendations should be prioritized. Moreover, the section does not address potential implementation challenges or barriers (e.g., a lack of digital literacy among farmers and infrastructure limitations in rural areas).

**Response:**

1.We sincerely thank the reviewer for the encouraging feedback and the useful suggestion. Following the recommendation, we have simplified the section titles in Main Conclusions and Policy Implications to make them more direct and clear.

2.We sincerely thank the reviewer for the insightful comment regarding the linkage between our contributions and the underlying theories. In response, we have thoroughly revised **Section 5.2 (Theoretical Contributions, page 26-27)** to explicitly connect our findings to the relevant theoretical frameworks.

Specifically, we have clarified how this study advances consumer behavior theory by demonstrating that customer decision-making in livestream marketing is dynamic and interaction-driven, highlighting the importance of real-time interaction and emotional resonance as identified in the Introduction. We also explicitly link the mediating role of customer satisfaction to user value theory and the value-satisfaction causal framework, showing how social presence functions as an upstream value driver and extends the applicability of these theories to interactive digital marketing contexts.

Moreover, we have strengthened the connection to trust theory by conceptualizing trust as a contextual climate rather than a dyadic relationship. We explain how the trust climate moderates both the direct and indirect effects of social presence, thereby extending trust theory to account for situational factors in high-uncertainty livestream marketing environments, particularly for agricultural products.

We believe these revisions more clearly demonstrate how our study builds on existing theories and advances understanding of consumer behavior in digital marketing contexts, as suggested.

3. We sincerely thank the reviewer for the valuable suggestions regarding the **Policy Recommendations**. In response, we have revised **Section 5.3 (page 27–29)** to rank the recommendations based on their importance and feasibility. Specifically, we have clarified why certain recommendations should be prioritized:
(1) **Promoting the development and standardization of a trusting atmosphere** is placed first, given that quality uncertainty in agricultural products makes a trust climate the most fundamental factor for sustained customer engagement.
(2) **Strengthening interactive mechanisms between livestream platforms and customers** is second, as high-quality real-time interaction operates most effectively on the foundation of trust.
(3) **Strengthen training and support for farmers and e-commerce platforms** is third, since competent actors are necessary for the successful implementation of a favorable trust climate and interactive mechanisms.
(4) **Promoting personalized and differentiated agricultural product livestream marketing** is fourth, as market segmentation and tailored strategies are most effective after the preceding policies are in place.

These revisions aim to make the policy recommendations more actionable, logically ordered, and practically grounded, as suggested.
